# A minimally-invasive serial cerebrospinal fluid sampling model in conscious Göttingen minipigs

**DOI:** 10.14440/jbm.2019.265

**Published:** 2019-01-11

**Authors:** Alessandra Bergadano, Eva Maria Amen, Björn Jacobsen, Sara Belli, Anthony Vandjour, Christelle Rapp, Claudia Senn

**Affiliations:** 1 Department for BioMedical Research, University of Bern, Murtenstrasse 35, CH-3008 Bern, Switzerland; 2 Roche Pharma Research and Early Development, Pharmaceutical Sciences, Roche Innovation Center Basel, F. Hoffmann-La Roche Ltd, Grenzacherstrasse 124, 4070 Basel, Switzerland

**Keywords:** CSF collection, Göttingen minipig, *in vivo* study, longitudinal CSF PK study, minimally-invasive model

## Abstract

Drug concentrations in cerebrospinal fluid (CSF) are typically used as a as a surrogate measure of their availability in the CNS, and CSF penetration in animal studies are used for assessment of CNS drug delivery in early preclinical drug development. The minipig is a valid alternative to dogs and non-human primates as non-rodent species in preclinical research, but this species presents anatomical peculiarities that make the serial collection of CSF technically challenging. A minimally-invasive serial cerebrospinal fluid collection model *via* catheterization of the subarachnoid space in conscious minipigs was developed allowing assessment of longitudinal drug pharmacokinetics in the central nervous system in preclinical research. Shortly, the subarachnoid space was accessed in the anesthetized minipig by puncture with a Tuohy needle; when CSF was flowing through the needle a catheter was advanced and thereafter tunneled and fixed on the back. The PK of peptide A administered subcutaneously was performed and CSF could be sampled in the conscious animals for up to 48 h. When compared to the plasma kinetic data, there was a clear difference in the elimination phase of Pept. A from CSF, with an apparent longer average terminal half-life in CSF. The 3Rs are addressed by reducing the number of animals needed for a pharmacokinetic profile in central nervous system and by improving the validity of the model avoiding biases due to anesthesia, blood contamination, and inter-individual variability.

## INTRODUCTION

In central nervous system (CNS) drug research, drug concentrations in cerebrospinal fluid (CSF) are typically considered a good surrogate of concentrations at pharmacological target site *e.g.*, the brain interstitial fluid (ISF), and are therefore used for pharmacokinetic/pharmacodynamics relationship assessment in both preclinical and clinical phases [1-3]. For drug candidates showing very low passive membrane permeability, *e.g.*, small CNS peptide analogues, their concentration and respective kinetic profile in brain and CSF cannot be predicted from their unbound plasma PK profile, neither from *in vitro* data, and must be obtained from *in vivo* animal studies. As first, rodent models are used and subsequently translated to larger animals. CSF can be sampled at the cerebral ventricles (CV), cisterna magna (CM) or lumbar thecal sites in conscious or anesthetized animals [4-6]. To perform a full PK profile of a compound in CNS, multiple CSF sampling time points are needed. Acute CSF models, with repeated puncturing with or without anesthesia or after euthanasia require a large number of animals for accommodating all time points, thereby posing a challenge to adhere to 3Rs and increasing inter-individual variability. Repeated puncturing is stressful for the conscious animal; manual puncture can result in blood vessel/tissue damage and consequent CSF contamination with blood as anesthesia can bias the experimental results. Chronic CSF sampling models try to circumvent these disadvantages by surgical implantation of a catheter into the brain or the lumbosacral space and bear the advantages of allowing repeated sampling of CSF in conscious, freely moving animals [7]. The minipig has become a valid alternative to dogs and non-human primates as non-rodent species in preclinical research [8,9]. Pigs have a stiff spine, massive vertebral bone structure with narrow intervertebral spaces (IVS), with the dorsal processes of the vertebrae tending to interfere with access to the IVS and a large bulk of epidural adipose tissue. These anatomical peculiarities make the serial collection of CSF technically challenging. In pigs, CSF is typically sampled from the CM [10], the CV [11] or *via* lumbar puncture under anesthesia or after euthanasia, with all disadvantages listed above. Spinal catheters for CSF collection could also be placed surgically by laminectomy [12]. Still this is invasive and requires advanced surgical skills that might not be available in each institution. We hypothesized that the listed technical limitations for CSF collection could be overcome by developing a minimally-invasive method *via* catheterization of the subarachnoid space allowing for serial CSF collection in conscious minipigs. The aim for the study presented was both to describe a minimally invasive method for CSF sampling through catheterization of the subarachnoid space and to validate this method within a pharmacokinetic study for a compound under investigation as therapeutic agent in CNS related disorders.

## MATERIALS AND METHODS

### Animals

All animals were Göttingen Minipigs purchased at Ellegaard Göttingen Minipigs A/S, Dalmose, Denmark (*n* = 3, female, aged 25–37 months, BW 43.7–52.2 kg). Animals were individually housed in an Association for Assessment and Accreditation of Laboratory Animal Care International (AAALACi) accredited facility with olfactory, visual, and auditory contact to their conspecifics in pens sized 8.6 m^2^, under a 12 h light, 12 h dark cycle. The environment was temperature-controlled (18°C ± 2°C) with relative humidity ranging from 40%–80%. Animals were offered commercially available laboratory swine diet (Provimi-Kliba, Kaiseraugst, Switzerland) twice a day and tap water ad libitum. Apart from a straw box for each animal, enrichment devices (chewing toys, balls, kongs) were offered on a rotational basis. All animals underwent an acclimatization phase of at least three weeks before study start.

### Use of experimental animals

The experimental protocol was approved by the cantonal veterinary authorities (Basel City) and all experiments performed according to the Swiss regulations and as described in the permission.

### Intraoperative anesthesia, analgesia, and monitoring

For catheterization, anesthesia was induced by using 8% sevoflurane (Sevorane^®^, AbbVie AG, Baar, Switzerland) with a flow of 2 L/min (1:1 air/O_2_) administered *via* a leak proof face mask, using a Matrx VMS^®^ anesthesia machine with a circle breathing system and a Tec7 Sevoflurane vaporizer. As soon as consciousness was lost, sevoflurane was reduced to 2%–4% and the carrier gas flow was set to 0.5–1 L/min (air/O_2_ = 50%/50%) for the duration of catheterization. A local anesthetic was injected subcutaneously (0.5 ml Lidocain 2%, Streuli Pharma AG, Uznach, Switzerland) at the insertion site to guarantee perioperative analgesia. Animals were covered with the warming blanket of a forced air warming device (Bair hugger^®^ model 750, 3M Switzerland Ltd., Rüschlikon, Switzerland) to ensure temperature close to the physiologic body temperature of 37°C–38°C in conscious Gottingen minipigs [13]. Eye ointment (Bepanthen^®^, Bayer AG, Zürich, Switzerland) was applied over the cornea to avoid desiccation. Heart rate (HR) and oxygen saturation of blood (SpO_2_) were continuously monitored *via* a pulse oximeter (Nonin Palm^®^ Model SAT 2500A Vet, Nonin Medical B.V. Europe, Amsterdam, Netherlands) in addition to rectal body temperature.

### Preparation

**Figure 1.**
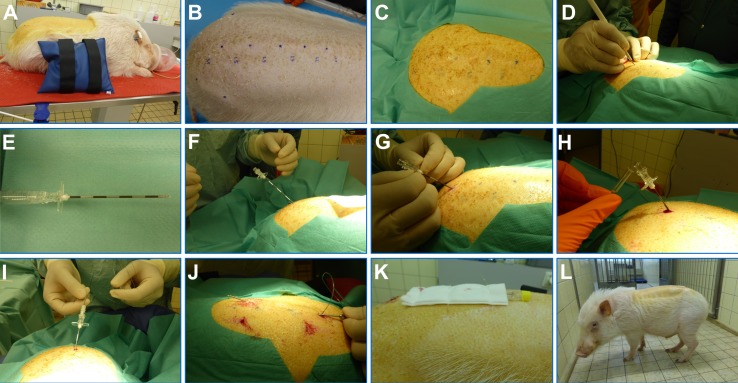
Procedure of catheterization of the subarachnoid space. **A.** Positioning of the anesthetized minipig in sternal recumbency, with the hind limbs stretched cranially; **B.** Highlighting of anatomical landmarks with each black dot representing the IVS; **C.** Aseptic preparation of surgical field and draping; **D.** Initial skin incision; **E.** Epidural needle with Tuohy bevel; **F.** Orientation and angle of epidural needle; **G.** Advancement of epidural needle (hanging drop technique); **H.** Free flow of CSF; **I.** Insertion of catheter through epidural needle; **J.** Subcutaneous tunneling of the catheter; **K.** Catheter with the closed sampling port and gauze compresses sutured to skin; **L.** Minipig after recovery from anesthesia with CSF catheter on the back.

Animals were placed in either sternal or lateral recumbency, with the hind limbs stretched cranially in order to widen the IVS (**Fig. 1A**). The IVS cranial to the last lumbar vertebra was chosen as insertion point and was identified with anatomical landmarks (**Fig. 1B**). Briefly, the two tubera coxae were palpated and marked with a waterproof marker. The lumbosacral intervertebral space was identified just caudal to an imaginary line drawn between both tubera coxae, indicated by a depression in the spinous processes. All spinous processes and IVS cranial to the last lumbar vertebra were marked. These landmarks are valuable for identifying the targeted IVS without imaging techniques or when using pigs with a higher amount of fatty tissue. The lumbar area was clipped and thoroughly washed and disinfected with iodine soap (Betadine^®^ soap, Mundipharma Medical Company, Basel, Switzerland). The skin was sprayed with alcoholic iodine solution (Betaseptic^®^, Mundipharma Medical Company, Basel, Switzerland) and covered in sterile self-adhesive surgical drapes (Foliodrape^®^, IVF Hartmann AG, Neuhausen, Switzerland) (**Fig. 1C**).

### Spinal catheterization

Catheterization was performed under aseptic conditions. An incision of about 5 mm was made in the skin above the intervertebral space cranial to the last lumbar vertebra with a disposable scalpel (#11, B.Braun, Aesculap AG, Tuttlingen, Germany) (**Fig. 1D**). An epidural needle (Perican^®^ Needle with Tuohy bevel, 18 G 1.3 × 80 mm; part of the Perifix^®^ 421 epidural anesthesia set, B. Braun Medical AG, Sempach, Switzerland) (**Fig. 1E**) was advanced with a ventro-cranial orientation forming an angle of 45° with the spinal cord through the skin, subcutaneous tissue, and interspinous ligament towards the subarachnoid space (**Fig. 1F-1G**). Entry into the epidural space was determined using either the loss-of-resistance (LOR) technique or the hanging drop (HD) technique. With the HD technique, the needle was filled with sterile saline and advanced until an inward movement of the saline was observed, as sign of the negative pressure present in the epidural space. With the LOR technique, a syringe filled with air was attached to the epidural needle, and loss of resistance to injection of air was examined intermittently while slowly advancing the needle, with clear loss of resistance identifying the epidural space. The needle was then carefully advanced further 5–10 mm into the subarachnoid space until CSF backflow was seen, indicating correct intrathecal positioning (**Fig. 1H**). Thereafter the stylet was removed and the epidural catheter (Perifix^®^ ONE, part of the described set) was fed through the epidural needle and gently advanced cranially in the subarachnoid space. Segmental markings on the catheter allowed determining that its tip was advanced approximately 8 cm into the subarachnoid space (**Fig. 1I**). This length was chosen to place the catheter at the level of the 1^st^ lumbar vertebra for study related sampling. Patency of the catheter was assessed by viewing the free CSF flow. The external part of the catheter was tunneled subcutaneously to add stability by using a small trocar to exit around 6 cm cranial to the insertion point (**Fig. 1J**). The external tip of the catheter was connected to Snap catheter connector (part of the described Perifix^®^ set). The skin incisions made prior to entry of the needle and for tunneling were adapted using non-absorbable monofilament suture material (Prolene^®^ 3-0, Johnson & Johnson Medical, Spreitenbach, Switzerland). Thereafter the catheter was covered with gauze compresses then both, catheter and compresses were sutured to the skin (**Fig. 1K**).

### Recovery and post-catheterization monitoring

Post-catheterization, sevoflurane administration was discontinued and the animals were visually supervised until recovery (**Fig. 1L**). Extrapolating from man the expected intensity of pain for this intervention, no post interventional analgesia was given. The minipigs were visually checked twice daily for integrity of the protective dressing and catheter and wellbeing. Action and endpoints were defined based on the recognition of clinical signs indicative of spinal cord impairment, neurologic deficit, pain or infection, and animals were monitored for 48 h post-catheterization using the clinical scoring system (**Table 1**). Depending on the severity, intervention consisted either in close monitoring for up to 48 h if signs did not get worse or cutoff with euthanasia.

### CSF sampling

The first CSF sampling began 24 h after catheter placement. At each time point, CSF was collected in two aliquots (100 µl each) under clean conditions by plunging the Snap connector with a sterile syringe and then applying gentle suction. The first sample withdrawn of CSF remaining in the catheter dead space was discarded. Sampling occurred in the home pen, restraining the minipig against one wall.

### Pharmacokinetic study design

**Figure 2.**
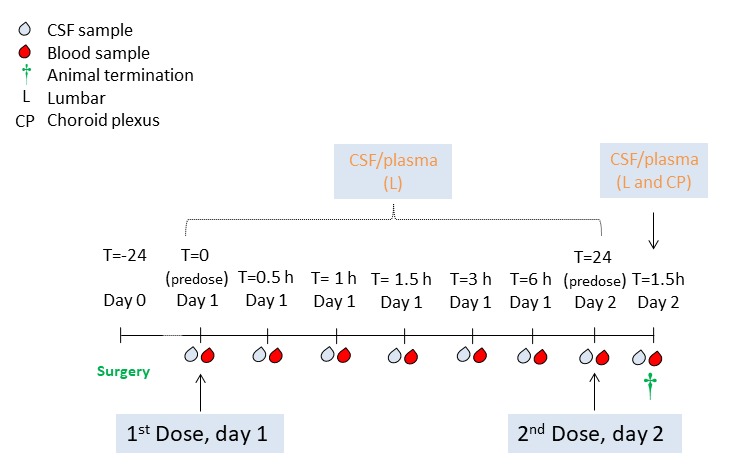
PK study layout. Pept. A was administered subcutaneously to three CSF catheterized animals.

Catheterization (*n* = 3) was performed on the day before the longitudinal CSF PK study (**Fig. 2**) as described above. Pept. A, a highly hydrophilic cyclic small peptide with a molecular weight (MW) of about 1 kDa, was administered twice at 4.2 mg/kg subcutaneously on study day 1 and again on study day 2, with a dosing interval corresponding to 24 h. Based on results of previous pharmacokinetic studies in rats, Pept. A was known to be able to reach the CSF after peripheral administration. Blood (100 µl) and CSF (100 µl) aliquots for drug concentration assessment were collected pre-dose, on study day 1 at 0.5, 1.5, 3, 6, and 24 h post first dose, and on study day 2 at 1.5 h post the second dose (corresponding to 25.5 h from study start) (**Fig. 2**). All blood samples were collected *via* microsampling (Microvette 100K3E, Sarstedt AG&Co, Nümbrecht, Germany) from the lateral ear vein because blood sampling from the jugular vein would have required placing the animals on their backs and thereby potentially compromising the patency of the CSF catheters. CSF was sampled as described above. On Study day 2, last samples collection was performed 1.5 h after the second administration of Pept. A (**Fig. 2**). Thereafter, animals were euthanized under anesthesia [8% sevoflurane (Sevorane^®^, AbbVie AG, Baar, Switzerland) by intravenous administration of an overdose of pentobarbital (120 mg/kg, Esconarkon^®^, Streuli Pharma AG, Uznach, Switzerland]. Gross and histopathological examination of the spine was performed to confirm the position of the catheter and evaluate potential changes of the spine. Samples were fixed in 10% buffered formalin (BioGnost d.o.o., Zagreb, Croatia), routinely embedded in paraffin (VIP^®^ 5, Sysmex, Horgen, Switzerland; Tissue-Tek^®^ TEC™ 5, Sakura Finetek Germany GmbH, Staufen, Germany), and 5 µm slides sections were cut (Microtome HM 350 S, Microm International GmbH, Walldorf, Germany) and deparaffinised, and routinely stained with hematoxylin eosin stain (Continous line stainer COT20, Medite GmbH, Burgdorf, Switzerland). EDTA-containing plasma was prepared from the collected blood samples by centrifugation; both CSF and plasma were stored at **−**20°C until bioanalysis. Bioanalytical assessment of Pept. A concentration in plasma and CSF matrices was performed by Liquid chromatography–mass spectrometry (LC-MS/MS).

In brief, the quantification of Pept. A levels in plasma and CSF was accomplished by means of LC MS/MS. Plasma and CSF were precipitated in four volumes of a mix of Ethanol and Acetonitrile (50:50 v/v) containing Bosentan (50 ng/ml) as internal standard, mixed and centrifuged (10 min, 3°C, 5889 g). Thereafter, 50 µl of the supernatant was diluted 50:50 on-line with [90% H_2_O + 9.8% Methanol + 0.2% Formic Acid v/v/v], then injected onto a Hypersil Gold 5 µM 10 × 2.1 mm Javelin Guards column washed with [90% H_2_O + 9.8% Methanol + 0.2% Formic Acid v/v/v] at 900 µl/min flow rate. After 48 s, the analyte and the internal standard were transferred on a Supelco Ascentis Express C18 2.1 × 20 mm 2.7 µM operating with mobile phase [H_2_O + Acetonitrile + Formic Acid v/v/v] at 600 µl/min flow rate. The outlet of the column was coupled to AB Sciex Qtrap 5500 mass spectrometer with TurboIonSpray source. Detection was carried out using multiple reactions monitoring mode with positive ion detection focusing in the transitions 989.26/687.20 for Pept. A.

Due to the small animal number, no statistical analysis was performed, but data were presented as mean values with standard deviation (SD) and coefficient of variation (CV%) calculated using Microsoft Excel 2010.

## RESULTS

Catheterization (*n* = 3) was performed on the day before the longitudinal CSF PK study (**Fig. 2**): Anesthesia was uneventful with HR between 52 and 88 beats per minute (BPM) for subject #220613 and 102 to 110 BPM for subject #222445, SpO_2_ was stable between 95 and 100% for both animals, and sevoflurane was kept at 4 Vol% for both animals. The anesthesia record of subject #219514 was missing.

The landmark based identification of the subarachnoid space was the most delicate part of the procedure. A maximum of 3 attempts were allowed for identifying the subarachnoid space or if blood was noticed. In this case the procedure was aborted and the minipig recovered. When the subarachnoid placement was confirmed by free flow of CSF in the epidural needle, the catheter could be advanced cranially very smoothly, and the free retrograde flow of CSF in the catheter was immediately visible. Typically, it took between 35 and 50 min for the intervention for catheter placement, counting from anesthesia induction until stopping sevoflurane administration.

Recovery from anesthesia was smooth and animals returned to their normal behavior and activities within 1h after recovery (**Fig. 1**). Serial CSF could be sampled in the conscious animals with minimal or no restraint; the samples were clear and transparent, thus not visibly contaminated with blood.

**Figure 3.**
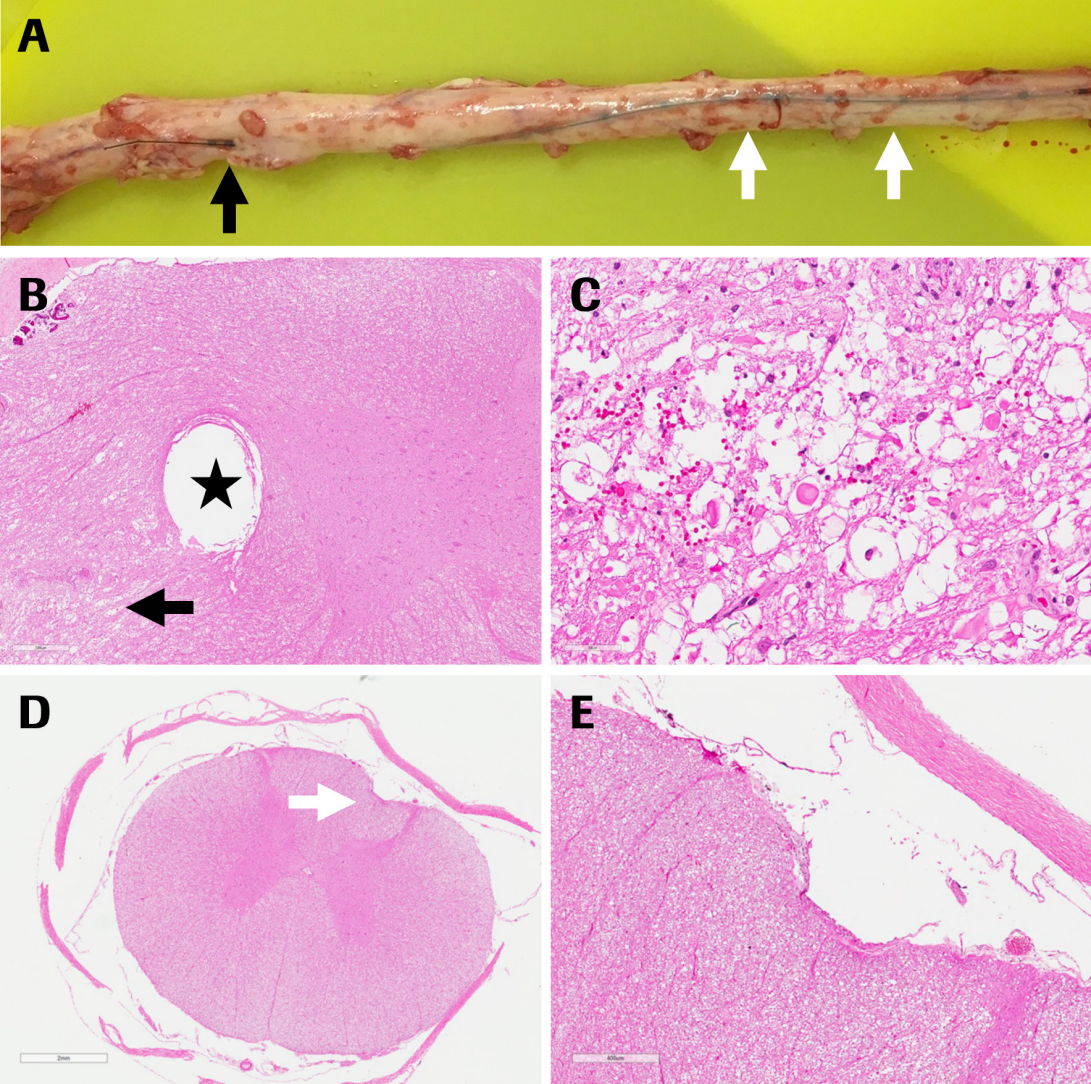
Pathological findings in subject #220613 with clinical signs. **A.** Spinal cord with insertion of catheter into parenchyma (black arrow) and cranial to the insertion dorsal presence of catheter (white arrows); **B.** Imprint of catheter (asterisk) and focal acute hemorrhage with associated spheroids (axonal degeneration) in ventrolateral region, black arrow points to region of degeneration; **C.** Higher magnification of (B) with hemorrhage and axonal degeneration; **D.** Lumbar spinal cord cranial to the insertion of the catheter, small imprint on dorsal spinal cord without parenchymal reaction (white arrow); considered secondary to fixation of tissue with catheter; **E.** Higher magnification of (D).

On study day 1 (24 h after catheter placement), subject #220613 showed mild monolateral ataxia of the right hind limb, without reaching cutoff criteria. Details of the pathological changes in the symptomatic minipig are shown in **Figure 3** and are explained by the insertion of the catheter in the spinal parenchyma distal to the insertion point. No gross and histopathological findings were present in the spinal cord of the two asymptomatic minipigs and their catheters were positioned dorsally.

**Figure 4.**
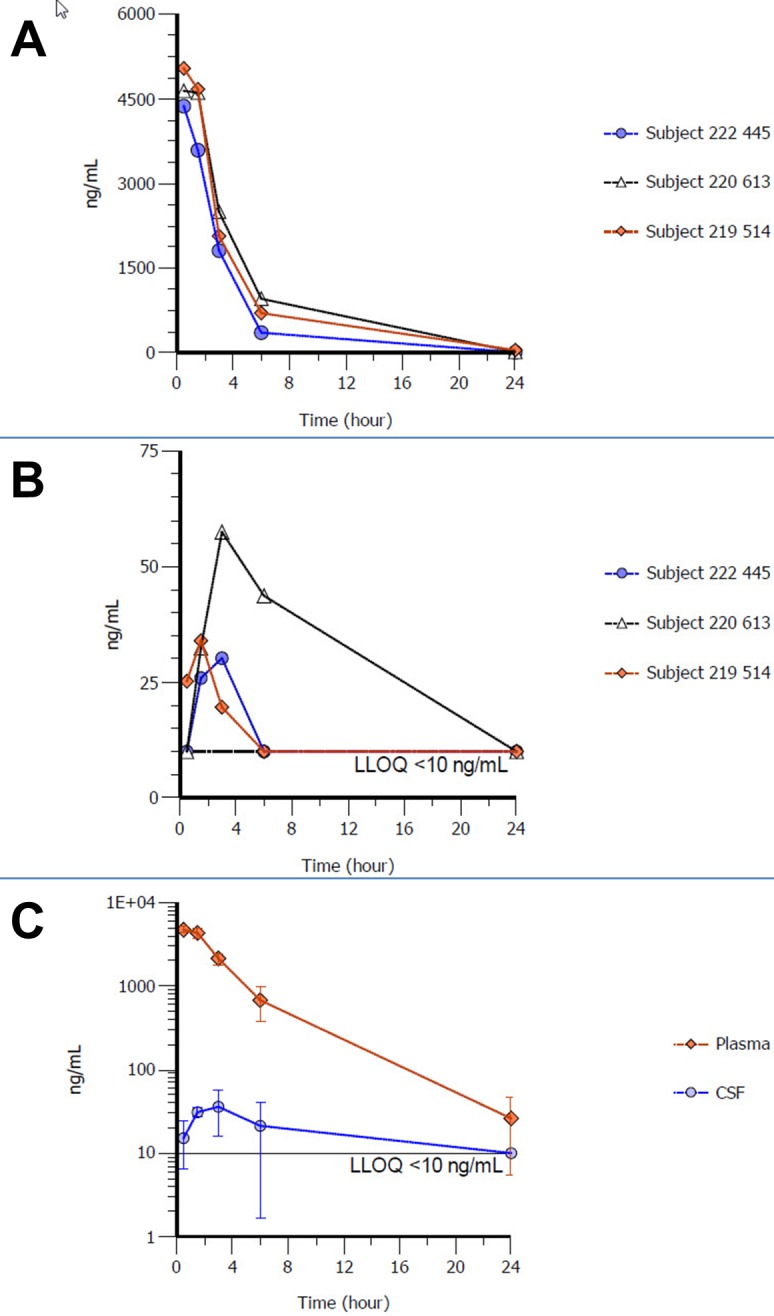
Concentration profile of Pept. A in CSF and plasma obtained from longitudinal sampling in minipigs after the first subcutaneous dose. **A.** Individual concentrations of Pept. A in plasma; **B.** Individual concentrations of Pept. A in CSF; **C.** Average (SD) concentrations of Pept. A in plasma and CSF The lower limit of quantification is also displayed.

The compound tested in the PK validation study was a small cyclic peptide with a MW of ca. 1 kDa, here named “Pept. A”, under investigation as therapeutic agent against a CNS-related disorder. Due to company policy and restrictions on disclosure of confidential information, its molecular structure and target cannot be reported here. The plasma and CSF concentrations and PK data are reported in **Figure 4** and in **Table 2** and **Table S1**. Pept. A was rapidly distributed in the circulation after SC injection, with an average Tmax of 0.5 h and short (3.5 h) derived average terminal half-life. In CSF, Pept. A concentration was above the lowest detection limit (> 10 ng/ml) until 24 h post first dose. When compared to the plasma kinetic data, there was a clear difference in the elimination phase of Pept. A from CSF, with an apparent longer average terminal half-life in CSF in agreement with previous observations in rats and estimated to be driven by the CSF flow rate. Regarding the uptake of Pept. A in the brain (CSF) from the circulation, an average AUC_0__-__24_CSF/plasma value of ca. 2% was calculated, explained by the limited passive permeability of the peptide and its hydrophilic nature. Despite of similar PK profiles obtained in plasma between animals, subject #220613 showed a remarkably higher Pept. A exposure in CSF. This observation could not be further explored in a follow-up work, but was considered to be related to the intactness of the Blood-Brain-Barrier or Blood-CSF-Barrier, likely lower in this animal, and/or to a more efficient active brain uptake of Pept. A. Finally, the exposure of Pept. A in the CSF at 1.5 h after the second dosing obtained from lumbar catheterization and choroid plexus (CP) sampling was comparable, indicating a homogenous distribution of the test compound in the CSF (**Table 2**).

## DISCUSSION

To the best of our knowledge this is the first description of minimally-invasive serial CSF sampling in conscious minipigs. The IVS cranial to the last lumbar vertebra was chosen for needle insertion, as it is the largest facilitating access [14]. When defining the anatomical landmarks, it has to be kept in mind that minipigs have a varying number of lumbar vertebrae (L5–L6) [14]; we recommend orientating from the lumbosacral IVS in cranial direction. Also the caudal IVS was chosen to reduce the risk of iatrogenic lesions to the spinal cord. Nevertheless, the conus medullaris in minipigs, as observed from the gross pathology, extends to sacral vertebra S2–S3 [15], making the risk for iatrogenic lesions higher than in other species. Strict adherence to standard technique for localizing the epidural space (loss of resistance or hanging drop) followed by advancing the needle 5 mm deeper is essential for a correct targeting of the dorsal subarachnoid space and avoidance of spinal cord damage. Intervertebral ligaments in swine are highly resistant hindering smooth insertion. The major technical limit remains the reliable identification and atraumatic access to the subarachnoid space. Prospectively the use of imaging techniques such as ultrasound, fluoroscopy or CT to improve the localization of the epidural and subarachnoid space should be investigated, enhancing success rate and safety.

The rationale for selecting an off-label epidural catheter instead of a spinal catheter was based on the larger lumen facilitating retrieval of CSF; spinal catheters of the necessary size are not commercially available. Another important technical feature of the chosen epidural catheter is its 3 lateral openings avoiding adhesion of its tip to the arachnoid membranes and consequent occlusion while sampling. The use of a large catheter in the narrow spines can lead to compression of the spinal cord clinically resulting in neurological deficits as observed in one animal (**Fig. 3**); therefore, the smallest size of epidural catheter allowing free flow of CSF should be selected.

Sevoflurane was chosen for anesthesia as it is safe and well tolerated in pigs for mask induction [16] and also for its favorable pharmacokinetic profile [17], with minimal metabolism and accumulation in fat which is proportional to the length of anesthesia. Based on these properties, the short anesthesia duration for the instrumentation, the 24 h wash-out period before first dosing and sampling in conscious animals, we did not expect any influence of the anesthetic on kinetics of the compound under investigation. This was a rationale in developing the model and its strength.

From previous *in vitro* essays (data not shown), it was known that Pept. A displayed a very low membrane passive permeability (below quantification) as well as no binding to plasma proteins. In rodents, systemic clearance of Pept. A was very high as expected due to the nature of the molecule, resulting in a very short terminal half-life in plasma < 0.5 h. The compound quickly reached the CSF after subcutaneous dosing and the kinetic in CSF was clearly different from those observed in plasma, suggesting that the plasma kinetic data of Pept. A could not be used to predict its exposure in the brain, and that measurements of longitudinal CSF PK profile in large animals were needed to progress the project into clinical trials. In minipigs, Pept. A was rapidly distributed in the circulation after SC injection, in line with the expectations for a small peptide showing no binding to plasma proteins, and aligned with previous PK data acquired in rodents. Regarding the uptake of Pept. A in the brain (CSF) from the circulation, the very low AUC_0__-__24_CSF/plasma value was aligned with data from previous rodent PK studies (AUC_0__-__24_CSF/plasma 1%–1.5%) where a CSF sample was collected at animal termination from CP site, increasing the confidence of human CSF PK translations.

In conclusion, we have described a new method refining serial CSF sampling in conscious minipigs. This technique is highly valuable for measuring the longitudinal CNS PK profile of drug candidates targeting the brain as it allows minimally-invasive determination of drug concentration at target site (CSF) in a time-dependent manner from the same animal. The availability of compound concentration kinetic at target site is essential for understanding the pharmacodynamic effect, for enabling drug candidate optimization and selection, and for allowing extrapolation of preclinical PK/PD data to human during early drug research phases. The described methodology reduces the number of animals, enhances PK data reliability by excluding inter-individual differences compared to a composite PK profile, and avoids technical biases of anesthesia or blood contamination. Although the approach is technically challenging, it is minimally invasive and well tolerated by the minipigs without disturbing their normal behavior. Overall it can be concluded that the 3Rs concept was successfully addressed and animal welfare improved.

## Supplementary Material

Supplementary information **Table S1**. Individual and average PK raw data obtained in minipig plasma and CSF.
Supplementary information of this article can be found online at
http://www.jbmethods.org/jbm/rt/suppFiles/265.

## Figures and Tables

**Table 1.** Action and end point criteria.

Symptoms	Decision	Actions
Paresis or mild ataxia of the hind limbs, mono- or bilateral Minipigs are able to walk, but are weak and wobbly in the rear legs. They may cross their back legs when walking, splay out, knuckle over or stumble in their back legs. Bladder and intestinal function is maintained.	Acceptable for max 48 h	Monitoring
Worsening of paresis to paralysis or from monolateral to bilateral Minipigs are still able to move their legs and wag their tails, but not strong enough to support its own weight and walk. Bladder and intestinal function is maintained but need to be supported.	Cut off	Euthanasia
Pain or fever Shivering, crying, muscle spasms, and/or a tense abdomen	Cut off	Removal of the spinal catheter and systemic treatment (antibiotics and analgesics)

**Table 2.** Average PK parameters ± SD (CV%) for Pept. A estimated in minipig plasma and CSF.

^a^PK parameters of Pept. A after SC administration based on plasma or CFS concentration data	Units	Plasma mean ± SD (CV%)	CSF lumbar mean ± SD (CV%)	CSF CP
Apparent terminal half-life (T1/2)	h	3.5 ± 0.7 (21%)	14.3 ± 5.5 (40%)	-
T_max_	h	0.5	3.0	-
C_max_	ng/ml	4683 ± 340 (7.2%)	35.8 ± 19 (54.7%)	-
AUC_0__-__24_	h*ng/ml	20970 ± 4820 (23%)	445 ± 244 (55%)	-
AUC_0__-__24_ CSF/plasma ratio	%	-	2.1	-
Mean Concentration at 25.5 h (1.5 h after the second dose)	ng/ml	4020 ± 643 (16%)	38.1 ± 18 (49%)	48.0^b^
CSF/plasma concentration ratio at 25.5 h (1.5 h after the second dose)	%	-	0.9 (range 0.4–1.5)	1.2

^a^PK parameters were estimated by non-compartmental analysis using CSF and plasma concentration data (*n* = 3 animals) with Phoenix 64.

^b^These data are from only one animal as the CSF sample collected from the other two subjects was visibly contaminated with blood, thus not usable. Data from Roche Study No. EX16_401380.
